# Melanin Metabolism: A Novel Oxidative Degradation Mechanism and Regulation by Hydrolyzed Conchiolin Protein

**DOI:** 10.1111/jocd.70770

**Published:** 2026-02-25

**Authors:** Xinyi Zhao, Haifeng Zeng, Long Zhu, Lihao Gu

**Affiliations:** ^1^ R&D Department Osman Biological Co., Ltd. Deqing China; ^2^ Beauty Hi‐Tech Innovation Co., Ltd. Kobe Japan

**Keywords:** hydrolyzed conchiolin protein (HCP), hydroxyl radicals, intracellular melanin degradation, keratinocytes, lysosomal proteolysis, melanin metabolism, oxidative degradation

## Abstract

**Background:**

Most pharmacological depigmenting agents and cosmetic skin‐brightening products achieve their effects by suppressing melanogenesis. However, the fate of melanin after melanosome transfer to keratinocytes—and the mechanisms governing its intracellular clearance—remains insufficiently explored.

**Aims:**

This study aimed to elucidate the intracellular mechanism of melanin degradation in keratinocytes and to establish a simplified and operable experimental strategy for evaluating melanin clearance beyond melanogenesis inhibition.

**Methods:**

A simplified in vitro model was established in which human epidermal keratinocytes phagocytosed isolated melanosomes, allowing investigation of melanin degradation independent of melanocyte activity. In parallel, a cell‐free oxidative system consisting of ferrous ions and hydrogen peroxide was employed to chemically induce hydroxyl radical–mediated melanin degradation. Lysosomal activity, intracellular oxidative status, hydroxyl radical (•OH) generation, melanin content, and pH dependence were assessed using fluorescence imaging and biochemical assays.

**Results:**

Keratinocytes exhibited a two‐step melanin degradation process involving lysosomal proteolysis followed by oxidative breakdown mediated by •OH. Treatment with hydrolyzed conchiolin protein (HCP) enhanced melanin degradation by promoting lysosomal activation and modulating intracellular oxidative conditions. Fluorescence imaging demonstrated partial colocalization of •OH signals with lysosomes and suggested alterations in lysosomal pH following HCP exposure. Chemical assays further revealed that alkaline conditions more effectively promoted hydroxyl radical–mediated melanin degradation.

**Conclusions:**

This study identifies an intracellular melanin degradation pathway operating within keratinocytes and presents a simplified experimental framework integrating cellular and cell‐free models. HCP emerges as a modulator of lysosomal–oxidative pigment clearance, offering an alternative pigmentation control strategy beyond melanogenesis inhibition and supporting the development of skin‐brightening approaches that preserve physiological pigment homeostasis.

## 1 Introduction

Melanin, the principal pigment responsible for human skin color, plays dual roles by determining pigmentation and providing photoprotection against ultraviolet (UV) radiation. Beyond this classical view, the skin is increasingly recognized as a complex neuro–immuno–endocrine organ that actively senses and responds to environmental stressors, including ultraviolet radiation. Ultraviolet exposure not only regulates local melanocyte activity but also modulates cutaneous immune responses and systemic neuroendocrine signaling, positioning pigmentation as part of a broader homeostatic adaptation rather than an isolated cellular event [1, 2, 3, 4]. It is synthesized within melanocytes and packaged into melanosomes, which are then transferred to surrounding keratinocytes. There, melanin accumulates above the nucleus, forming supranuclear caps that shield genomic DNA from UV‐induced damage [4, 5, 6, 7]. In addition to photoprotection, accumulating evidence suggests that melanin and melanogenic intermediates influence intracellular redox balance, inflammatory signaling, and cellular stress responses, underscoring the multifaceted biological roles of pigmentation [8]. This process—melanogenesis, encompassing both synthesis and intercellular transfer—has been extensively studied and forms the basis of our current understanding of skin pigmentation [9, 10].

Melanogenesis itself is a highly regulated and context‐dependent process, controlled by a complex network of ultraviolet radiation, neuroendocrine mediators, inflammatory signals, and intracellular redox states. Classical and contemporary reviews have demonstrated that melanin synthesis is not solely governed by tyrosinase activity, but is dynamically modulated at transcriptional, post‐translational, and organelle levels in response to environmental and physiological cues. Moreover, melanin and its biosynthetic intermediates exert diverse biological effects, including antioxidant buffering, pro‐oxidant activity under specific conditions, metal ion chelation, and immune modulation. This functional diversity highlights that melanogenesis is not intrinsically pathological, but rather an adaptive process whose dysregulation—or inappropriate suppression—may compromise cutaneous homeostasis [3, 11, 12, 13].

In contrast, the mechanisms underlying melanin degradation and clearance within keratinocytes remain less clearly defined. As keratinocytes differentiate and migrate toward the stratum corneum, intracellular melanin content gradually decreases. While this reduction has traditionally been attributed to passive dilution during epidermal turnover, emerging evidence suggests that active intracellular processes, particularly lysosome‐dependent degradation, also contribute significantly [14, 15, 16, 17]. Compared with the extensive investigation of melanogenesis and melanosome transfer, post‐transfer melanin clearance within keratinocytes has received disproportionately limited attention [3, 14, 15, 16, 17].

Melanosomes exhibit lysosome‐like features, including an acidic lumen and the presence of hydrolytic enzymes such as Cathepsin L2, which is capable of degrading melanosomal membranes [15]. However, the fate of the exposed melanin core after membrane breakdown is still unclear [18]. Given melanin's inherent chemical stability and resistance to enzymatic degradation, non‐enzymatic mechanisms, such as oxidation by reactive oxygen species (ROS), have been proposed. Notably, lysosomes can produce hydroxyl radicals (•OH), the most reactive form of ROS, which may play a role in melanin oxidation and clearance [19, 20]. Nevertheless, the physiological involvement of ROS in intracellular melanin metabolism remains to be fully elucidated.

To investigate this, a simplified in vitro model was established in which human keratinocytes phagocytose isolated melanosomes, thereby mimicking melanin accumulation independent of melanocyte‐derived stimuli. This system provides a controllable platform for interrogating keratinocyte‐driven pigment turnover, which is particularly relevant to conditions in which melanogenic stimulation has subsided but residual pigmentation persists, such as post‐inflammatory hyperpigmentation or photoaging‐associated dyschromia.

Within this context, hydrolyzed conchiolin protein (HCP)—a nacre‐derived bioactive ingredient known for its skin‐brightening effects—was selected for further analysis. Previous studies have shown that HCP can suppress melanogenic signaling pathways, including those mediated by endothelin‐1 (ET‐1), α‐melanocyte‐stimulating hormone (α‐MSH), and microphthalmia‐associated transcription factor (MITF), as well as tyrosinase activity [21, 22, 23, 24]. Such mechanisms represent the most widely adopted skin‐brightening strategies in cosmetic science, as the majority of depigmenting agents aim to reduce visible pigmentation by directly or indirectly suppressing melanogenesis. These approaches are effective in attenuating melanin overproduction and have been extensively validated in both experimental and clinical contexts [25, 26, 27]. However, accumulating evidence suggests that melanogenesis is a highly complex and context‐dependent biological process, regulated by diverse intracellular pathways and environmental stimuli, and that melanin itself exerts multiple physiological functions beyond pigmentation. As a consequence, long‐term or indiscriminate suppression of melanogenic activity may not fully align with the dynamic homeostasis of cutaneous pigmentation, highlighting the need to explore complementary strategies that operate downstream of melanin synthesis [3, 11, 12].

The present study investigated a two‐step degradation mechanism involving lysosomal proteolysis followed by ROS‐mediated melanin oxidation. By focusing on post‐transfer melanin metabolism within keratinocytes, this work aims to complement conventional melanogenesis‐centered strategies and to establish a physiologically compatible framework for regulating pigmentation without broadly suppressing melanin production. The regulatory effects of HCP on lysosomal activity, intracellular oxidative conditions, and melanin clearance were also evaluated. These findings may provide new insights into melanin metabolism and inform the development of intracellular pigmentation control strategies beyond conventional melanogenesis inhibition.

## 2 Materials and Methods

### 2.1 Assessment of HCP on Keratinocyte Proliferation

Hydrolyzed conchiolin protein (HCP) was prepared from nacre powder using a multi‐step process. Briefly, nacre powder was first subjected to acid hydrolysis to remove the calcium carbonate matrix and release the protein fraction. The recovered conchiolin fraction was then neutralized, thoroughly washed, and subjected to a secondary enzymatic hydrolysis step. The resulting hydrolysate was sequentially purified through centrifugation (to remove insoluble residues), ceramic membrane filtration (to eliminate high‐molecular‐weight impurities), and ultrafiltration (to remove unstable low‐molecular‐weight compounds). The final hydrolyzed product was obtained by freeze‐drying into a solid form.

For the proliferation assay, normal human epidermal keratinocytes (NHEKs, Kurabo Industries Ltd., Osaka, Japan) were seeded into 96‐well plates at 10000 cells/well in 99 μL of culture medium. HCP was diluted in PBS and added at 1 μL per well to achieve final concentrations of 1.0 to 0.00098 mg/mL. Controls received 1 μL of PBS. After 72 h of incubation at 37°C in 5% CO_2_, 10 μL of Cell Counting Kit‐8 reagent (Dojindo, Kumamoto, Japan) was added to each well. Absorbance at 450 nm was measured at 0 and 120 min using a multi‐detection microplate reader (PowerScan HT; DS Pharma Biomedical Co. Ltd., Osaka, Japan). ΔAbs (120 min) was used to evaluate cell proliferation. Experiments were performed in triplicate (*n* = 3).

### 2.2 Establishment and Analysis of an Intracellular Melanin Degradation Model

To investigate intracellular melanin degradation independent of melanocyte‐derived melanogenic signaling, an in vitro model was established in which keratinocytes phagocytosed isolated melanosome‐enriched fractions. This approach enables controlled evaluation of melanin turnover within keratinocytes without confounding effects from ongoing melanin synthesis or intercellular signaling.

#### 2.2.1 Preparation of Melanosome‐Enriched Fractions

B16 melanoma cells (Cell Resource Center for Biomedical Research, Institute of Development, Aging and Cancer, Tohoku University, Sendai, Japan) were cultured in 75 cm^2^ flasks until confluence. Culture medium was removed, and cells were detached using 5 mL of Trypsin–EDTA (Gibco, Thermo Fisher Scientific, Waltham, MA, USA) followed by incubation at 37°C for 20 min. The enzymatic reaction was neutralized with DMEM GlutaMAX supplemented with 10% fetal bovine serum (FBS), and cells were collected by centrifugation.

To disrupt cellular membranes and release intracellular organelles, the cell pellet was washed twice with phosphate‐buffered saline (PBS) and mechanically homogenized. The homogenate was subjected to differential centrifugation to enrich melanosome‐containing fractions. Briefly, the homogenate was centrifuged at 1000 rpm for 2 min to remove unbroken cells and large debris. The resulting supernatant was then centrifuged at 10000 rpm for 2 min to pellet melanosome‐enriched fractions. The final pellet was resuspended in 500 μL of keratinocyte culture medium for subsequent use.

#### 2.2.2 Phagocytosis of Melanosomes by Keratinocytes

Normal human epidermal keratinocytes (NHEKs) were incubated with the prepared melanosome suspension in 25 cm^2^ culture flasks and cultured at 37°C in a humidified atmosphere containing 5% CO_2_ for 24 h to allow efficient phagocytosis. This incubation period was selected based on preliminary observations confirming stable intracellular melanosome accumulation.

After phagocytosis, keratinocytes were detached using 0.05% Trypsin–EDTA and seeded into 96‐well plates at a density of 10 000 cells per well in 99 μL of keratinocyte culture medium.

#### 2.2.3 Evaluation of Intracellular Melanin Degradation

Hydrolyzed conchiolin protein (HCP) was diluted in PBS and added to the cultures at final concentrations of 6.25, 12.5, or 25 mg/mL by adding 1 μL per well. Control wells received 1 μL of PBS only. Cells were cultured for an additional 72 h to allow intracellular processing and degradation of phagocytosed melanosomes.

Following treatment, culture medium was removed, and cells were gently washed with PBS to eliminate extracellular residues. Intracellular melanin content was quantified by adding 50 μL of 1 mol/L NaOH to each well to solubilize melanin, followed by measurement of absorbance at 550 nm using a multi‐detection microplate reader (PowerScan HT, DS Pharma Biomedical Co. Ltd., Osaka, Japan).

To correct for background absorbance and ensure specificity, NHEKs that had not been exposed to melanosomes were processed in parallel under identical conditions and used as negative controls. Final intracellular melanin values were calculated by subtracting the absorbance of non–melanosome‐loaded keratinocytes from that of melanosome‐loaded cells.

#### 2.2.4 Microscopic Observation

For qualitative assessment of intracellular pigmentation, keratinocytes containing phagocytosed melanosomes were seeded onto poly‐L‐lysine–coated 35 mm glass‐bottom dishes (No. 1S, cover glass thickness 0.16–0.19 mm; Matsunami Glass Ind. Ltd., Osaka, Japan) and treated with HCP (12.5 or 25 mg/mL) for 72 h. Intracellular pigmentation was observed using an Olympus IX70 inverted phase‐contrast microscope (Olympus, Tokyo, Japan).

### 2.3 Immunofluorescence Analysis of Cathepsin L2 and Lysosomal Localization

NHEKs containing phagocytosed melanosomes were detached using 0.05% Trypsin–EDTA (5 mL) and incubated for 30 min at 37°C. Cells were seeded into poly‐L‐lysine‐coated 35 mm glass‐bottom dishes at 99 μL per dish and incubated for 24 h. HCP (12.5 or 25 mg/mL in PBS) was added at 1 μL per dish, and cells were cultured for 72 h. Controls received 1 μL of PBS only. After treatment, cells were fixed with 4% paraformaldehyde, permeabilized with 0.1% Triton X‐100, and blocked with 1% bovine serum albumin (BSA). Primary antibodies were applied: anti‐Cathepsin L2 (Alexa Fluor 546, Thermo Fisher Scientific, USA) and anti‐LAMP1 (Alexa Fluor 488, Thermo Fisher Scientific, USA). After washing with PBS, DAPI (4′,6‐diamidino‐2‐phenylindole; Thermo Fisher Scientific, USA) was used for nuclear staining. Fluorescence images were acquired using a confocal laser scanning microscope (FV3000, Olympus, Tokyo, Japan).

### 2.4 Investigation of the Oxidative Mechanism Underlying Melanin Degradation

#### 2.4.1 Intracellular Detection of ROS Induced by Hydrogen Peroxide

A 30% hydrogen peroxide solution (m.w. 34.01) was diluted with Milli‐Q water. Photo‐oxidation Resistant DCFH‐DA (Dojindo, Kumamoto, Japan) was dissolved in DMSO to prepare a 10 mmol/L stock solution, which was then diluted 1:1000 with Loading Buffer Solution to obtain a 10 μmol/L working solution. NHEK and HaCaT cells (purchased from CLS, Eppelheim, Germany) containing phagocytosed melanosomes were seeded into culture dishes suitable for confocal microscopy. After removing the medium, cells were washed with PBS and incubated with 200 μL of the DCFH‐DA working solution for 30 min at 37°C. Following incubation, the solution was removed, and cells were washed with PBS. Next, 2 μL of 10 mmol/L hydrogen peroxide was added to 2 mL of basal medium in each dish and incubated for 30 min. Cells were then fixed with 4% paraformaldehyde for 5 min. DAPI (diluted 1:500 in PBS) was added at 200 μL per dish and incubated for 5 min to stain nuclei, followed by PBS washes. Fluorescence and bright‐field images were acquired using a confocal laser scanning microscope (FV3000) under bright‐field, UV excitation, and blue excitation modes.

#### 2.4.2 Intracellular Detection and Localization of Hydroxyl Radicals

HPF (hydroxyphenyl fluorescein) was used to monitor •OH generation. HPF is a non‐fluorescent probe that becomes fluorescent upon reaction with highly reactive oxidative species, particularly •OH, due to oxidation‐induced restoration of its fluorescein conjugation system. Less reactive species such as hydrogen peroxide or superoxide do not efficiently activate HPF under physiological conditions, making it suitable for detecting hydroxyl radical‐dominant oxidative activity in our model [28].

NHEK cells containing phagocytosed melanosomes were stained with LysoTracker Red DND‐99 (100‐fold dilution in PBS, Thermo Fisher Scientific) and hydroxyphenyl fluorescein (HPF; 10‐fold dilution in PBS, Goryo Chemical Inc., Sapporo, Japan). Each reagent (10 μL) was added to 2 mL of culture medium, and cells were incubated at 37°C for 1 h. After staining, the medium was removed, and cells were fixed with 1 mL of 4% paraformaldehyde in phosphate buffer for 10 min at room temperature. After PBS washes, DAPI (200‐fold dilution in PBS) was added at 2 mL per dish and incubated for 10 min. Cells were washed again with PBS prior to imaging. Fluorescence images were acquired using a confocal laser scanning microscope (FV3000).

#### 2.4.3 Assessment of Hydroxyl Radical Generation Induced by HCP (Fluorescence‐Based)

The same staining procedure and imaging conditions described in Section 2.4.2 were used.

NHEK cells were treated with HCP at final concentrations of 0.05, 0.1, or 0.2 mg/mL, while control cells received PBS. After incubation with HPF and LysoTracker and subsequent fixation and DAPI staining as previously described, fluorescence images were acquired using a confocal laser scanning microscope (FV3000).

### 2.5 Evaluation of Lysosomal pH in Melanosome‐Phagocytosing Keratinocytes

NHEK cells containing phagocytosed melanosomes were cultured in 2 mL of medium. To assess lysosomal pH, cells were incubated with 2 μL of pHrodo Green AM Intracellular pH Indicator (Thermo Fisher Scientific) and 1 μL of LysoTracker Red DND‐99 for 30 min at 37°C. After staining, the medium was removed, and cells were fixed with 1 mL of 4% paraformaldehyde in phosphate buffer for 10 min at room temperature. Cells were then washed twice with PBS. DAPI (200‐fold dilution in PBS) was added at 2 mL per dish and incubated for 10 min. After a final PBS wash, cells were observed using a confocal laser scanning microscope (FV3000). The effect of hydrolyzed conchiolin protein (HCP, 0.1 mg/mL) on melanolysosomal pH was assessed using the same procedure.

### 2.6 Assessment of Melanin Degradation Induced by Hydroxyl Radicals

To evaluate the capacity of •OH to induce melanin degradation under controlled chemical conditions, an in vitro Fenton reaction system was employed. This system was designed to independently verify the oxidative degradation of melanin observed in cellular experiments.

#### 2.6.1 Fenton Reaction Setup

Hydroxyl radicals were generated via a classical Fenton reaction using hydrogen peroxide (H_2_O_2_) and ferrous ions (Fe^2+^). Ferrous sulfate (FeSO_4_) was used as the Fe^2+^ source. Unless otherwise stated, all reactions were performed in the presence of H_2_O_2_.

Each reaction was carried out in a 96‐well plate with a total volume of 100 μL per well. The reaction mixture contained hydrogen peroxide, Fe^2+^, and either melanin or hydroxyphenyl fluorescein (HPF), which served as a fluorescence probe for •OH generation.

#### 2.6.2 pH‐Dependent Analysis

To examine the influence of pH on •OH generation and melanin degradation, reactions were conducted in phosphate buffer adjusted to pH 3.0, 5.7, 7.0, 8.7, or 11.0. Each well contained H_2_O_2_ at a final concentration of 10 mM, Fe^2+^ at a final concentration of 1 mM, and melanin or HPF at the indicated concentrations.

After incubation at room temperature for 60 min, melanin degradation was assessed by measuring absorbance at 550 nm using a multi‐detection microplate reader (PowerScan HT, DS Pharma Biomedical Co. Ltd., Osaka, Japan). In parallel, HPF fluorescence intensity was recorded to estimate •OH generation.

#### 2.6.3 Fe^2+^ Concentration–Dependent Analysis

To evaluate the effect of Fe^2+^ availability on •OH production and melanin degradation, Fe^2+^ was added at increasing concentrations while maintaining the reaction pH at 7.0. Hydrogen peroxide concentration was kept constant throughout the experiment.

Following 60 min of incubation at room temperature, melanin absorbance at 550 nm and HPF fluorescence intensity were measured as described above.

### 2.7 Statistical Analysis

All experimental data are presented as mean ± standard deviation (SD). Statistical analyses were performed using unpaired *t*‐tests with Microsoft Excel. Differences were considered statistically significant at *p < 0.05 and highly significant at **p < 0.01.

## 3 Results

### 3.1 Effect of HCP Concentration on Keratinocyte Proliferation

**FIGURE 1:**
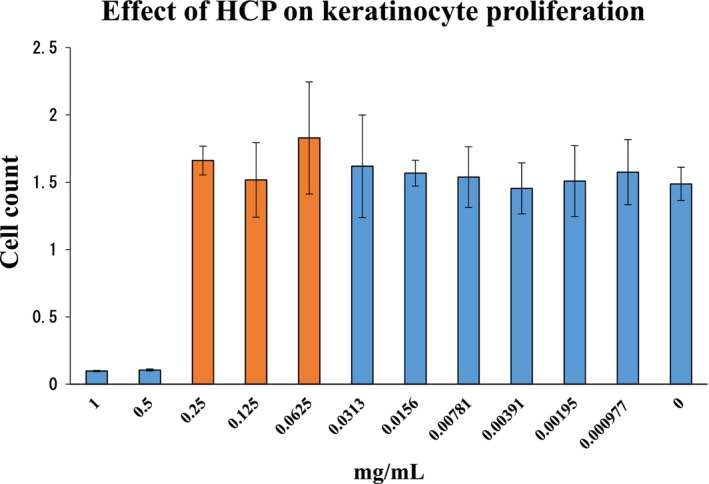
Effect of hydrolyzed conchiolin protein on keratinocyte proliferation. The results show that HCP concentrations up to 0.25 mg/mL did not affect cell viability, whereas ≥ 0.5 mg/mL led to a moderate reduction in proliferation. Based on these findings, we selected ≤ 0.25 mg/mL for all subsequent experiments to avoid potential cytostatic effects. Data are presented as mean ± SD (*n* = 3).

To validate the experimental conditions for subsequent melanin degradation studies, the effect of HCP on keratinocyte proliferation was first evaluated. Cell viability remained comparable to the control at concentrations up to 0.25 mg/mL. In contrast, concentrations of 0.5 mg/mL and above were associated with a reduction in proliferation. These observations support the use of HCP at concentrations ≤ 0.25 mg/mL in downstream assays to avoid potential cytostatic interference (Figure 1).

### 3.2 Quantitative and Visual Assessment of Intracellular Melanosome Complex Degradation

**FIGURE 2:**
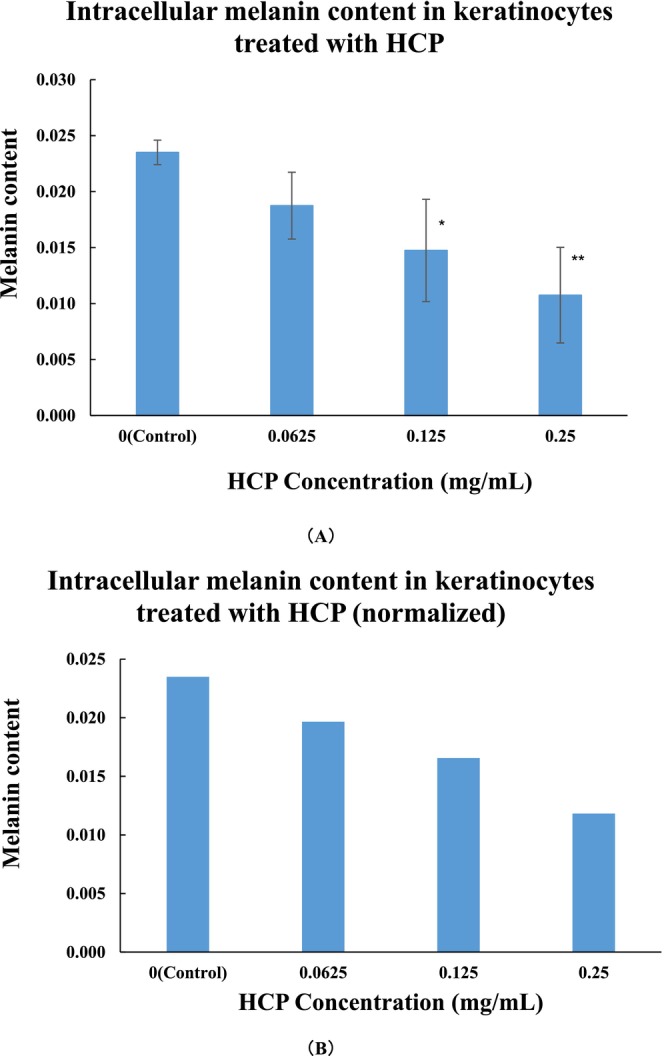
Intracellular melanin content in keratinocytes treated with hydrolyzed conchiolin protein (HCP). (A) Quantification of intracellular melanin based on absorbance at 550 nm after 72 h of HCP treatment; (B) Melanin content normalized to cell number to account for potential variations in cell proliferation. Data are presented as mean ± SD (*n* = 4). *p < 0.05, **p < 0.01 vs. Control (PBS).

**FIGURE 3:**
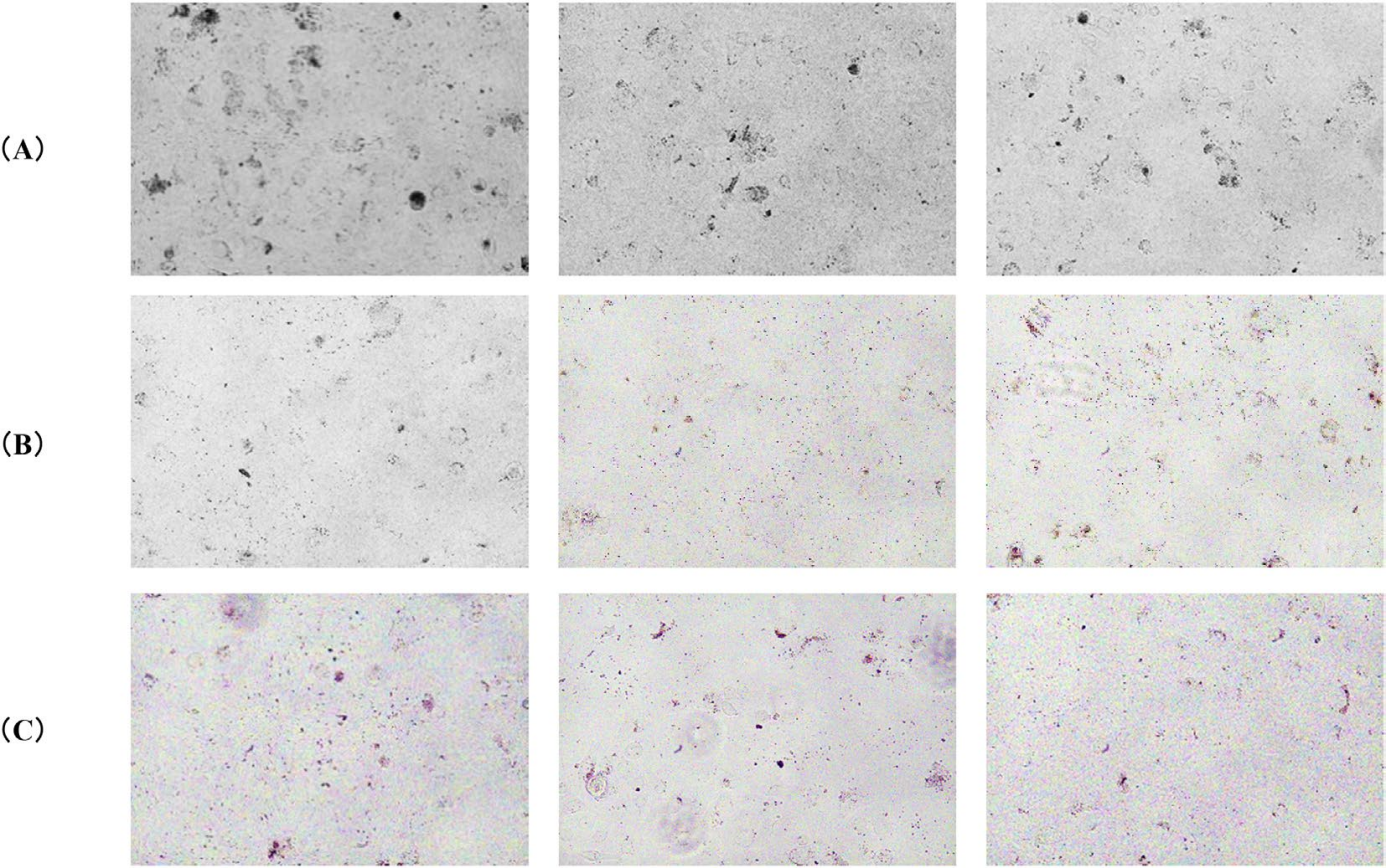
Phase‐contrast imges showing intracellular pigmentation in keratinocytes. (A) 0 mg/mL (Control); (B) 0.125 mg/mL HCP; (C) 0.25 mg/mL HCP. Reduced pigmentation was observed in HCP‐treated cells, consistent with the quantitative results.

To determine whether HCP enhances the intracellular degradation of melanosome complexes in keratinocytes, intracellular melanin content was quantified by measuring absorbance at 550 nm. Treatment with HCP at 0.125 mg/mL and 0.25 mg/mL significantly reduced melanin levels compared to the control group (Figure 2A, *p < 0.05, **p < 0.01 vs. Control). Normalization to cell number confirmed that this reduction was not attributable to differences in proliferation (Figure 2B). These results indicate that HCP promotes the intracellular degradation of melanin‐containing organelles in keratinocytes. Based on these quantitative results, effective concentrations of HCP (0.125 and 0.25 mg/mL) were subsequently applied in a separate experiment for phase‐contrast microscopy. After 72 h of treatment, cells exposed to HCP exhibited lighter overall pigmentation compared to the untreated control (Figure 3), providing visual support for intracellular melanin degradation.

### 3.3 Immunofluorescence Observation of Cathepsin L2 and Lysosomal Colocalization

**FIGURE 4:**
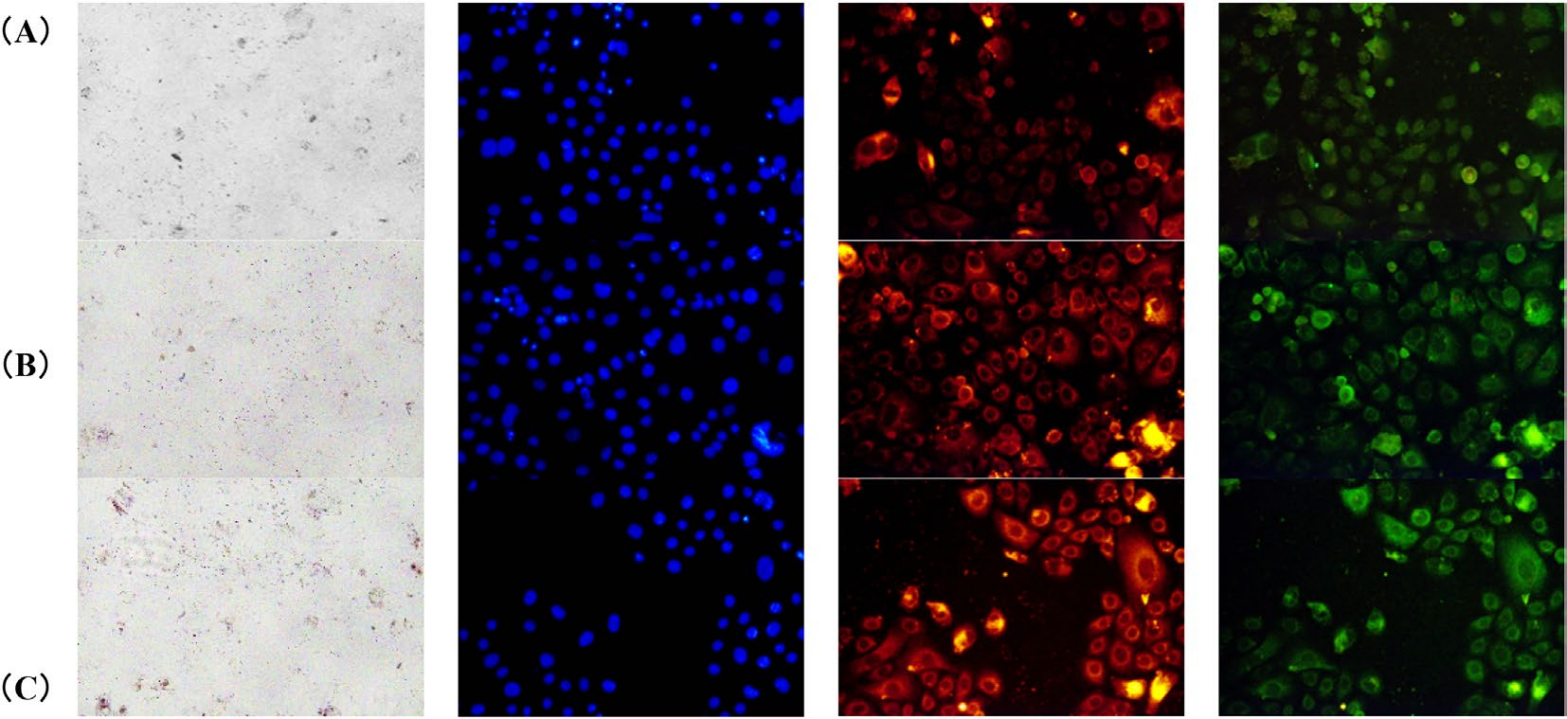
Representative immunofluorescence images of keratinocytes treated with hydrolyzed conchiolin protein (HCP) at 0, 0.125, and 0.25 mg/mL. From left to right, each column shows bright‐field images (pigmentation), DAPI staining (nuclei, blue), Cathepsin L2 (red), and LAMP1 (green). (A) 0 mg/mL (Control); (B) 0.125 mg/mL HCP; (C) 0.25 mg/mL HCP. HCP treatment increased Cathepsin L2 expression and colocalization with LAMP1, indicating enhanced lysosomal activity.

To evaluate whether HCP influences lysosomal degradation of melanosome complexes, immunofluorescence staining was conducted to assess the expression and colocalization of Cathepsin L2 and the lysosomal marker LAMP1. In untreated keratinocytes, Cathepsin L2 was detected at a basal level with limited overlap with LAMP1. Following HCP treatment at 0.125 mg/mL and 0.25 mg/mL for 72 h, Cathepsin L2 fluorescence intensity increased, accompanied by enhanced colocalization with LAMP1. These observations indicate that lysosomal processing of melanosome complexes was promoted under HCP treatment (Figure 4).

### 3.4 Intracellular ROS and Hydroxyl Radical Localization in Melanosome‐Phagocytosing Keratinocytes

**FIGURE 5:**
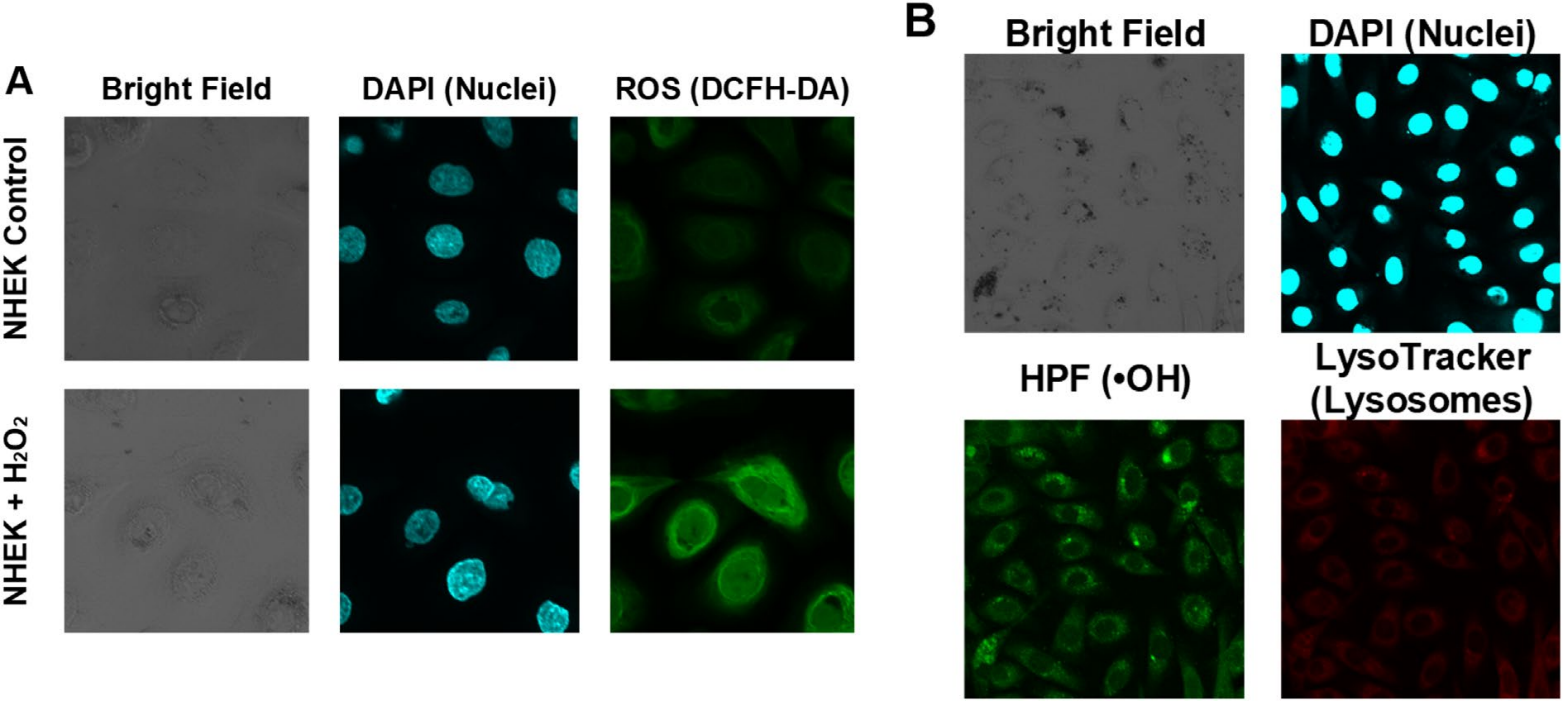
Intracellular visualization of oxidative species in NHEK cells phagocytosing melanosomes. (A) NHEK cells treated with or without H_2_O_2_ (10 mmol/L). Images show: Bright field (melanosomes), DAPI (nuclei, blue), and ROS detection using DCFH‐DA (green). (B) NHEK cells stained for hydroxyl radicals and lysosomes. Images show: Bright field (melanosomes), DAPI (blue), HPF (•OH, green), and LysoTracker (lysosomes, red).

To investigate oxidative mechanisms potentially contributing to melanin degradation, the intracellular localization of reactive oxygen species (ROS) and •OH was examined in NHEK cells containing phagocytosed melanosomes. Cells were treated with hydrogen peroxide (H_2_O_2_, 10 mmol/L), and ROS distribution was visualized using DCFH‐DA staining. Compared to untreated controls, H_2_O_2_‐treated NHEKs showed increased ROS fluorescence distributed throughout the cytoplasm. These signals did not colocalize with melanosome‐containing structures observed under bright‐field microscopy (Figure 5A). In HaCaT cells, ROS fluorescence was minimal under the same conditions, even with increased confocal sensitivity (data not shown). In a separate experiment, •OH were detected using hydroxyphenyl fluorescein (HPF). HPF signals partially overlapped with LysoTracker staining, indicating the presence of •OH in lysosome‐associated regions containing melanosomes (Figure 5B).

### 3.5 Assessment of Hydroxyl Radical Signals and Lysosomal pH in HCP‐Treated Keratinocytes

**FIGURE 6:**
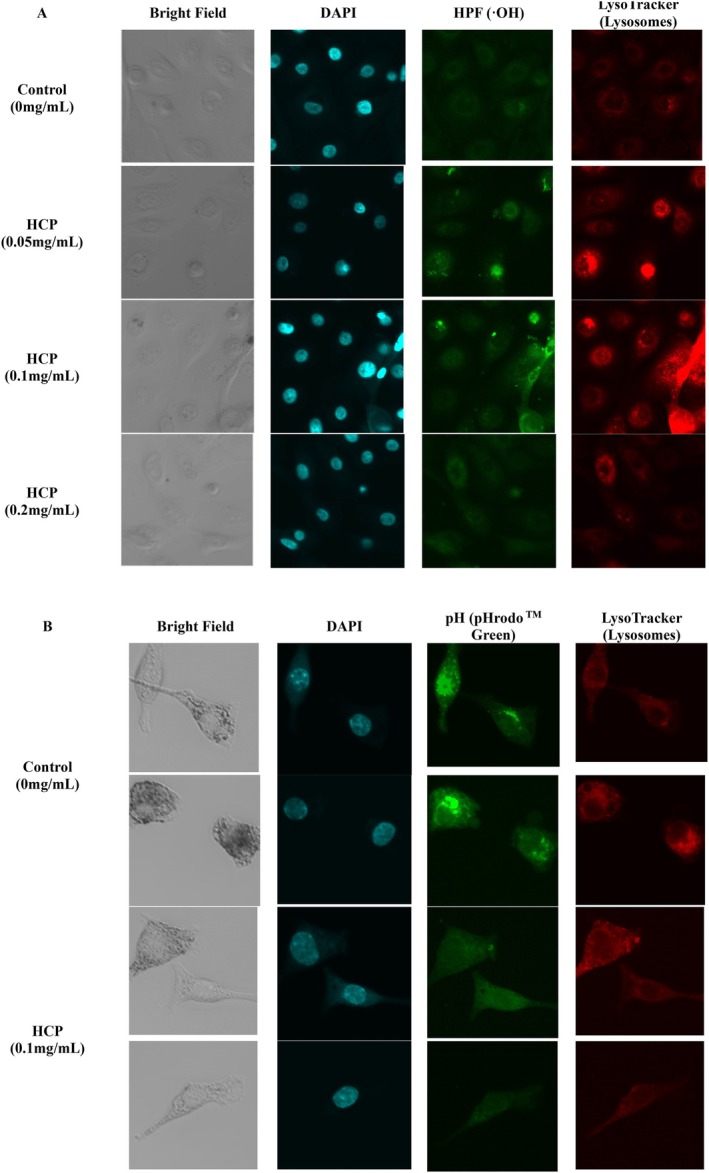
Intracellular observation of hydroxyl radicals and lysosomal pH in HCP‐treated keratinocytes. (A) NHEK cells treated with HCP (0–0.2 mg/mL) were stained with DAPI (blue), HPF (•OH, green), and LysoTracker (red). Increased HPF signals were observed at 0.05 and 0.1 mg/mL, partially overlapping with lysosomes. (B) pHrodo Green AM staining shows reduced fluorescence after 0.1 mg/mL HCP treatment, indicating a potential shift in lysosomal pH. Bright field and DAPI are shown for reference.

To investigate the effects of HCP on the intracellular oxidative microenvironment, fluorescence‐based assays were performed to assess •OH distribution in keratinocytes that had phagocytosed melanosomes. Cells were treated with HCP at 0.05, 0.1, and 0.2 mg/mL, and fluorescence signals for HPF (•OH indicator) were examined. Increased HPF intensity was observed at 0.05 and 0.1 mg/mL, while no apparent elevation was noted at 0.2 mg/mL (Figure 6A). The HPF signals partially overlapped with LysoTracker staining, indicating that •OH were localized within lysosome‐associated compartments.

To further assess the lysosomal environment, pHrodo Green AM staining was used to evaluate pH changes. pHrodo Green is a pH‐sensitive fluorogenic dye whose fluorescence is inversely correlated with environmental alkalinity. At neutral to alkaline pH, the dye predominantly adopts a non‐fluorescent, closed spirolactone structure, resulting in minimal fluorescence. Upon exposure to acidic conditions, protonation induces a conformational transition to an open, π‐conjugated form, leading to a marked increase in fluorescence intensity. Consequently, pHrodo Green becomes dimmer in alkaline environments and brightly fluorescent in acidic compartments, making it particularly suitable for monitoring endosomal and lysosomal acidification in cellular systems. Treatment with 0.1 mg/mL HCP resulted in a visibly decreased fluorescence intensity compared to the control (Figure 6B), suggesting a potential shift toward a less acidic lysosomal pH under this condition.

### 3.6 In Vitro Evaluation of Hydroxyl Radical–Induced Melanin Degradation

To investigate the role of •OH in melanin degradation under controlled chemical conditions, an in vitro Fenton reaction system was employed. Reactions were performed at varying pH levels and Fe^2+^ concentrations to assess both •OH generation and melanin degradation.

**FIGURE 7:**
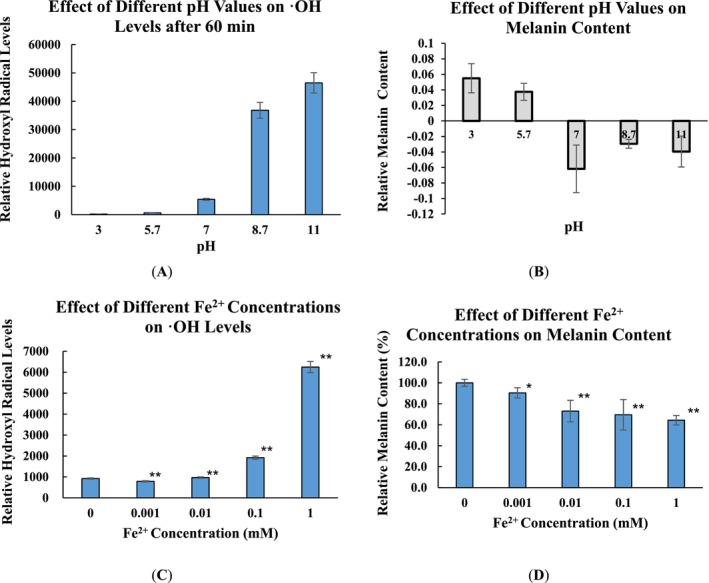
In vitro assessment of hydroxyl radical–mediated melanin degradation. Hydroxyl radicals were generated by a Fenton reaction containing Fe^2+^ and hydrogen peroxide (H_2_O_2_). (A) Effect of different pH values on ·OH levels after 60 min. (B) Effect of different pH values on melanin content. (C) Effect of Different Fe^2+^ Concentrations on ·•OH Levels at pH 7. (D) Effect of Different Fe^2+^ Concentrations on Melanin Content at pH 7. Data are presented as mean ± SD (*n* = 4). *p < 0.05, **p < 0.01 versus Control (PBS).

In the pH‐dependent assay, HPF fluorescence increased progressively from pH 7.0 to 11.0, indicating enhanced •OH production under alkaline conditions (Figure 7A). Correspondingly, melanin absorbance at 550 nm decreased with increasing pH (Figure 7B), suggesting pH‐dependent degradation of melanin.

To examine the effect of Fe^2+^ concentration, FeSO_4_·7H_2_O was added at increasing levels while maintaining the pH at 7.0. Melanin absorbance decreased in a concentration‐dependent manner (Figure 7C), and HPF fluorescence intensity increased in parallel (Figure 7D), indicating that higher Fe^2+^ availability promoted •OH generation and subsequent melanin degradation.

## 4 Discussion

Traditionally, skin‐brightening strategies have focused on inhibiting melanogenesis through three primary mechanisms: (1) suppression of tyrosinase activity, a rate‐limiting enzyme in melanin synthesis; (2) inhibition of melanogenic signaling pathways (e.g., MITF, α‐MSH, ET‐1); and (3) blocking the transfer of melanosomes from melanocytes to keratinocytes [25]. These approaches are effective in treating hyperpigmentation disorders such as melasma and lentigines [26, 27], where melanin overproduction presents a clinical issue.

However, applying such strategies broadly to healthy skin may carry biological risks. Certain phenolic or catecholic tyrosinase inhibitors and their metabolites have been associated with chemical leukoderma [29, 30, 31]. Despite the availability of in vitro and in vivo assays for evaluating safety [32, 33], their limitations in physiological relevance warrant continued caution. Furthermore, melanin plays a vital role in shielding keratinocyte nuclei from UV‐induced DNA damage [5]; indiscriminate suppression of its synthesis or transfer may impair photoprotection, increasing vulnerability to photoaging and post‐inflammatory hyperpigmentation [34, 35].

From a broader physiological perspective, melanogenesis and melanin accumulation are integral components of the skin's adaptive response to environmental stressors rather than merely cosmetic determinants. The skin functions as a neuro–immuno–endocrine organ that integrates ultraviolet radiation, oxidative stress, inflammatory mediators, and hormonal signals to maintain local and systemic homeostasis. Within this framework, melanogenesis is dynamically regulated in response to ultraviolet exposure, immune activation, and metabolic cues, serving protective, redox‐buffering, and immunomodulatory roles. Consequently, melanin synthesis is not inherently pathological, and its indiscriminate suppression may interfere with essential adaptive processes [1, 2, 3, 4, 5, 6, 7].

Comprehensive reviews have demonstrated that melanogenesis is governed by a multilayered regulatory network extending far beyond tyrosinase activity alone. These regulatory mechanisms include transcriptional and epigenetic control, organelle biogenesis, intracellular trafficking, redox signaling, and crosstalk with neuroendocrine and inflammatory pathways. Moreover, melanin and its biosynthetic intermediates exert diverse biological effects, including antioxidant buffering, pro‐oxidant activity under specific conditions, metal ion chelation, and modulation of immune responses. This functional diversity underscores that pigmentation must be tightly balanced rather than globally suppressed to preserve cutaneous homeostasis [3, 8, 9, 10, 11, 12, 13].

In this context, strategies that selectively modulate post‐transfer melanin handling within keratinocytes—rather than broadly inhibiting melanogenesis—may offer a more physiologically compatible approach to pigmentation control. Targeting intracellular melanin degradation allows attenuation of excessive pigment accumulation while preserving the upstream adaptive functions of melanocyte activation and melanin synthesis.

This study identifies a previously underappreciated intracellular degradation mechanism mediated by lysosomal and oxidative pathways, presenting a novel strategy beyond melanogenesis inhibition. By isolating keratinocyte‐driven degradation in a simplified model, we demonstrate that HCP enhances both lysosomal proteolysis and oxidative breakdown of melanin.

Importantly, our model mimics a physiological state in which melanosomes, once phagocytosed, become biologically redundant due to the absence of environmental stimuli. This condition permits observation of intrinsic degradation pathways independent of UV exposure. Our findings confirm that lysosomal proteolysis is the initial step in melanosome degradation. Cathepsin L2, a key lysosomal protease [15, 36], was present in untreated cells and colocalized with LAMP1 after HCP treatment [37, 38], suggesting HCP enhances baseline lysosomal degradation capacity. This mechanism is also consistent with multiple previous studies reporting that lysosome‐mediated autophagy within keratinocytes contributes to melanosome degradation, thereby promoting the attenuation of pigmentation [16, 17, 39, 40]. Autophagic pathways have been shown to facilitate the breakdown of internalized melanosomes under certain conditions, supporting their role in skin tone regulation and pigment clearance.

These results confirm the involvement of lysosomal proteolysis in melanosome degradation. Following membrane disruption, melanin is further metabolized via oxidative pathways, highlighting a proteolytic–oxidative cascade for intracellular pigment clearance.

Following the proteolytic disruption of melanosomal membranes, further breakdown of exposed melanin requires oxidative processing. Given melanin's chemical stability, such degradation likely involves reactive oxygen species (ROS) [41], which are highly reactive chemical intermediates derived from oxygen metabolism and other redox processes. ROS encompass species such as hydrogen peroxide, superoxide, hydroxyl radicals, and singlet oxygen, and are known to participate in oxidative modifications of biological macromolecules under certain conditions.

From a chemical perspective, melanin exists as a dynamic redox system rather than a chemically uniform polymer. Its structure comprises a mixture of reduced and fully oxidized units, including quinone‐ and quinonimine‐type species, with a minor fraction of intermediate radical forms. These species are maintained in a redox equilibrium that arises from interactions between reduced and oxidized melanin subunits [3, 42, 43]. Importantly, such intrinsic redox heterogeneity provides a plausible chemical basis for melanin's susceptibility to highly reactive oxidative species, including •OH, once the polymer becomes accessible following melanosomal disruption. Previous studies by Sarna and co‐workers [44, 45] have systematically investigated the oxidative chemistry of eumelanin, demonstrating that reactive oxygen species—particularly hydroxyl radicals—can induce oxidative cleavage of indole rings, fragmentation of the polymer backbone, and loss of optical absorbance in the visible spectrum. These oxidative modifications are associated with changes in melanin redox properties and structural integrity, ultimately leading to functional degradation rather than simple bleaching.

In this context, the hydroxyl radical–mediated melanin degradation observed in the present study is chemically consistent with the mechanisms described by Sarna et al. Although the precise molecular intermediates were not characterized here, the observed decrease in melanin absorbance under hydroxyl radical–generating conditions strongly suggests oxidative disruption of the conjugated melanin structure. Importantly, our findings extend these classical chemical observations into a biologically relevant intracellular setting, where melanin degradation occurs following lysosomal membrane disruption and exposure to an oxidative microenvironment.

In our model, increased •OH in lysosomal regions was specifically monitored using hydroxyphenyl fluorescein (HPF). HPF is a non‐fluorescent probe under basal conditions, derived from a fluorescein scaffold in which the π‐conjugated system is chemically “caged”. Oxidative activation of HPF requires disruption of this caged structure through aromatic hydroxylation or oxidative cleavage—processes that demand exceptionally high redox potential and rapid reaction kinetics. Among common reactive oxygen species, •OH possess both the strongest oxidizing power and diffusion‐controlled reaction rates, enabling direct attack on aromatic rings and efficient restoration of the fluorescein conjugation system. In contrast, less reactive species such as hydrogen peroxide, superoxide, or singlet oxygen lack sufficient reactivity to induce these structural transformations under physiological conditions [28, 33, 46]. Consequently, HPF fluorescence preferentially reflects •OH–dominant oxidative activity rather than general ROS accumulation. Using this approach, we investigated •OH‐mediated degradation in a cell‐free Fenton reaction system [47] under varying pH conditions. The results showed that both HPF fluorescence and melanin absorbance decreased more significantly under acidic conditions, indicating that melanin oxidation by •OH is pH‐sensitive. These findings are consistent with classical Fenton chemistry [47, 48] and support a sequential model in which lysosomal proteolysis precedes oxidative degradation.

Although the precise mechanism remains to be elucidated, two possible scenarios can be proposed: (1) HCP may alter lysosomal microenvironmental parameters, indirectly facilitating •OH production; or (2) HCP‐induced disruption of melanosomal membranes may expose melanin to existing oxidative conditions, thus accelerating its breakdown. Notably, Cathepsin L2 functions optimally under acidic pH (3–5) [49], while melanosomes are typically more neutral (pH 5–7) [50, 51]. The observed decrease in pHrodo Green AM fluorescence following HCP treatment suggests a potential shift in lysosomal acidity, although no consistent dose–response was seen at higher concentrations (e.g., 0.2 mg/mL). This may reflect the technical limitations of capturing pH fluctuations in live cells rather than a true absence of effect. Indeed, the intracellular oxidative microenvironment is influenced by multiple parameters beyond pH, including metal ion availability and redox balance—factors not fully addressed in this study but important for future exploration.

These observations highlight the complexity of intracellular oxidative dynamics, particularly within lysosomal compartments, and support the need for further mechanistic investigation. While the current findings demonstrate that HCP facilitates melanin degradation via lysosomal and oxidative mechanisms, whether HCP actively regulates ROS generation or lysosomal pH remains to be determined. This represents a key limitation of the study, as well as a promising direction for future research. Nonetheless, our data robustly support the conclusion that melanin can be degraded intracellularly through a proteolytic–oxidative cascade initiated by keratinocytes.

**FIGURE 8:**
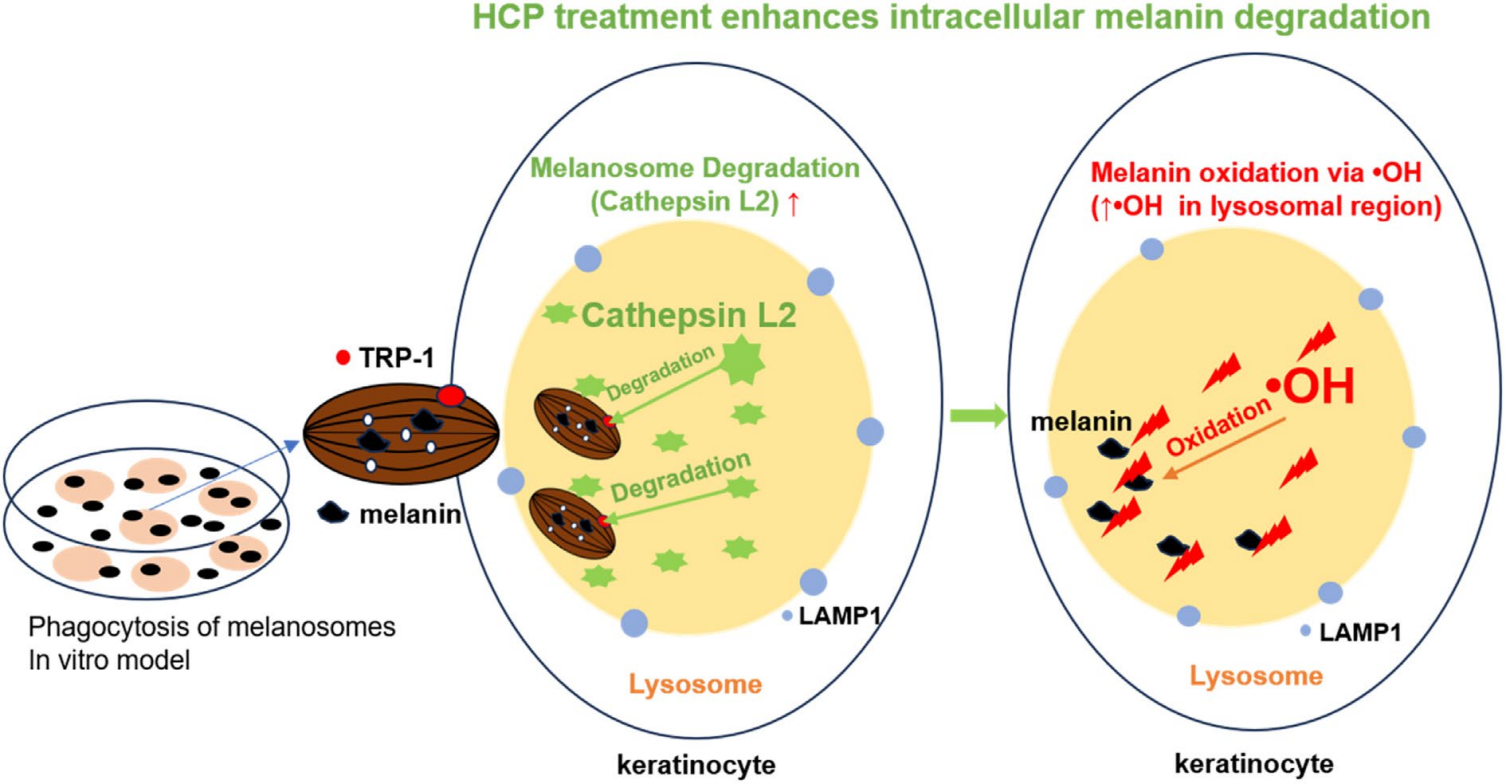
Proposed two‐step mechanism of intracellular melanin degradation enhanced by HCP: Lysosomal proteolysis followed by oxidative breakdown via hydroxyl radicals.

Collectively, these findings support a model in which HCP promotes melanin degradation through sequential proteolytic and oxidative processes within lysosomes (Figure 8).

Future studies should validate these mechanisms in in vitro 3D skin models or clinical settings to assess translational relevance. Investigating HCP's effects across skin phototypes may further elucidate individual differences in pigment clearance capacity. In addition, precision delivery systems may be developed to enhance melanin degradation. For instance, lysosome‐targeted nanoparticles encapsulating Fe^2+^ or Cu^2+^ ions could localize Fenton‐like activity within keratinocytes [52, 53]. Combined with agents like HCP, such approaches could activate dual proteolytic–oxidative pathways to achieve effective yet physiologically compatible brightening. Integration of these strategies into future formulations offers exciting potential for biologically intelligent cosmetic development.

## 5 Conclusion

This study elucidates a sequential mechanism of intracellular melanin degradation in keratinocytes, involving lysosomal proteolysis followed by •OH‐mediated oxidation. Using an in vitro melanosome phagocytosis model, we demonstrated that hydrolyzed conchiolin protein (HCP) enhances this degradation cascade by promoting lysosomal activity and modulating the intracellular oxidative microenvironment. While the exact pathways through which HCP facilitates •OH generation remain to be clarified, the data support its role as a facilitator rather than an initiator of melanin degradation.

These findings advance our understanding of pigment turnover beyond melanocyte‐centered melanogenesis, highlighting a keratinocyte‐intrinsic pathway with potential relevance for hyperpigmentation conditions. Future studies in physiologically relevant models are warranted to further define the molecular mediators involved and to explore translational applications that preserve pigment homeostasis while enhancing epidermal clarity.

## Author Contributions

Lihao Gu: conceptualization. Haifeng Zeng: methodology. Xinyi Zhao, Haifeng Zeng, and Lihao Gu: software. Xinyi Zhao and Lihao Gu: validation. Xinyi Zhao and Long Zhu: formal analysis. Lihao Gu: investigation. Long Zhu: resources. Haifeng Zeng: data curation. Haifeng Zeng: writing – original draft preparation. Xinyi Zhao and Lihao Gu: writing – review and editing. Haifeng Zeng: visualization. Long Zhu: funding acquisition. All authors have read and agreed to the published version of the manuscript.

## Funding

This work was supported by Osman Biological Co. Ltd.

## Ethics Statement

This study did not involve human participants or animals.

## Consent

The authors have nothing to report.

## Conflicts of Interest

The authors declare no conflicts of interest.

## Data Availability

The data that support the findings of this study are available from the corresponding author upon reasonable request.
